# Exploring Drug Resistance: Microbial Profiles, Antibiotic Sensitivity, and Biofilm Development in Orthopedic Implant Infections

**DOI:** 10.7759/cureus.70938

**Published:** 2024-10-06

**Authors:** Kristi Sarkar, Summaiya Mullan, Hari Menon

**Affiliations:** 1 Clinical Microbiology, Government Medical College and New Civil Hospital, Surat, IND; 2 Orthopedics, Government Medical College and New Civil Hospital, Surat, IND

**Keywords:** antibiogram, antibiotic, biofilm, implants, orthopedic, resistance

## Abstract

Background

With the advent of and rise in antibiotic resistance globally, especially in postoperative patients, studying the antibiogram and associated factors is the need of the hour. The present study was undertaken to document the microbiological profile in postoperative orthopedic patients with the infected implant in situ and to observe the antibiotic susceptibility patterns of isolated organisms in such infections.

Methods

This study was conducted in the Department of Microbiology of a tertiary care hospital for six months after obtaining institutional ethical approval. A total of 236 samples from patients with orthopedic implant infections were received during the study period, out of which 53 samples with positive culture isolate were further analyzed for microbiological profile including biofilm production. All observations and demographics were recorded and analyzed using SPSS software version 21.0 (IBM Corp., Armonk, NY, USA) and represented in the form of graphs, data, and tables.

Results and conclusion

The study showed a culture positivity rate of 53 (23%) out of 236 samples, in which gram-negative isolates 36 (68%) were more than gram-positive isolates 17 (32%). The most common isolate was *staphylococcus aureus* 17 (32%) of which the majority were MRSA 13 (76%), followed by *Escherichia coli* 9 (17%) and Klebsiella pneumoniae 9 (17%). Out of the 53 isolates, 20 were biofilm producers. Biofilm-producing isolates were more resistant to tested routine antibiotics compared to non-biofilm. This study could represent the initial interdisciplinary effort in an ongoing process to better understand and manage orthopedic implant infections at the hospital, specifically focusing on infections related to orthopedic devices.

## Introduction

Orthopedic surgeries took a turn when implants and prosthetics completely changed the treatment outcome of complicated fractures, joint dislocations, degenerative joint disease, etc [1]. Implants brought with them advantages like early bone recovery, decrease in pain and increase in mobility in patients, and improved life in quantity and quality [2]. However, orthopedic procedures still involve extensive instrumentation. As a result, a breach in the sterile environment is not uncommon, either a surgical opening or an already open wound or fracture [3]. It has been observed that implant infections complicate and alter the outcomes of orthopedic surgeries in almost 6% of cases in India leading to extended and expensive hospital stays for the patients [4].

The cause of these infections is multifactorial ranging from the nature of the implant to the type of pathogen involved. The source is often found to be from temporary bacteremia, an improperly sterilized instrument or linen, or bacteria present on the patient’s body or the environment at large. The most commonly associated species among the gram-positive isolates are *Staphylococcus aureus*, coagulase-negative staphylococci (CoNS), Streptococci, and Enterococci. Pseudomonas, Acinetobacter, and Enterobacterales are the commonly found gram-negative isolates. Anaerobes like Propionibacteria and Peptostreptococci account for the less commonly found isolates [5].

While at most tertiary care centers, surgeons provide the best medical and surgical precautions, prophylaxis, and treatment available, certain bacterial mechanisms are hard to avoid and penetrate. Biofilm production is one such mechanism that provides a fortress for the bacteria making the lives of the antibiotics very difficult. The three major adverse effects of biofilm formation are persistent bacterial infection, antibiotic resistance, and the generation of a chronic inflammatory response. Organisms in a biofilm are 1000-1500 times more resistant to antibiotics than in their planktonic state [6].

Antibiotic resistance has emerged as an urgent global public health threat causing millions of deaths every year worldwide [7]. The severity of the threat is such that microbes develop resistance against an antibiotic as early as two years into the market [8]. The rampant use of antibiotics has given birth to multidrug-resistant superbugs, the most notorious amongst them being the ESKAPE pathogens, such as *Enterococcus faecium, Staphylococcus aureus*, Klebsiella pneumoniae, *Acinetobacter baumannii, Pseudomonas aeruginosa*, and Enterobacter spp.

Many of these multidrug-resistant bacteria, such as MRSA, vancomycin-resistant *Staphylococcus aureus* (VRSA), multidrug-resistant Acinetobacter, extended-spectrum β-lactamase-producing Enterobacteriaceae (ESBLs), and multidrug-resistant* Pseudomonas aeruginosa*, have been linked to infections associated with orthopedic implants [9].

The diagnosis of orthopedic and trauma device-related infections (ODRIs) faces a number of challenges with repeated negative culture reports. This could be attributed to reasons like the low metabolic activity of bacteria from biofilm samples and preoperative and operative antibiotics. Similarly, the management of these patients becomes difficult owing to persistent and recurrent infections and terrifying antimicrobial resistance [10].

Regional studies contributing to the knowledge about the etiopathogenesis of ODRIs are very few creating a gap between cause and effect especially in tertiary care setups. Thus, the present study aimed to investigate the most prominent causative organisms and their structural and functional behavioral patterns in postoperative hosts in a tertiary care center in western India.

## Materials and methods

Study design

This is a cross-sectional prospective study that was conducted in the Department of Microbiology of a tertiary care hospital in Surat (Gujarat, India) for a period of six months from January to July 2023 after ethical approval by the Human Research Ethical Committee of the institute. Approval from the institutional human research ethics committee was obtained before the start of the study, under the ethical approval number. GMCS/STU/ETHICS-2/Approval/18974/23.

Inclusion criteria

Patients of all ages and sexes with implants and prostheses in situ, presenting with symptoms of local infection in the implant site whose samples are sent for microbiological investigation are included in the study.

Exclusion criteria

The repeat specimens of the same patient and specimens having polymicrobial flora were excluded from the study.

Methodology

A total of 236 samples including pus aspirate, swab, and tissue specimens were received in the microbiology laboratory. Among these, 53 samples of patients with infection at the implant sites showing positive culture isolates were included in the study. All demographic details were recorded and samples like pus aspirate, swabs, and tissue were analyzed for gram stain, Ziehl-Neelsen (ZN) stain, and KOH (10% potassium hydroxide) wet mount microscopies. All samples were plated in routine bacteriological culture media, given optimum conditions, and studied for growth. Isolated colonies were identified following guidelines given in CLSI M35-A2 for laboratory identification of bacteria and yeast. After identification, the isolates were subjected to antimicrobial susceptibility testing using the Kirby-Bauer disc diffusion test and the Epsilometer test. These tests were performed according to the standards provided in CLSI document number M100 2022 and M07. Detection of biofilm formation by the isolates was performed using the microtitre plate method and quantification of biofilm was done by reading the optical density using an ELISA reader.

Statistical analysis

All observations and demographics were recorded and analyzed using SPSS software version 21.0 and represented in the form of graphs, data, and tables. The normality of continuous data was tested using the Shapiro-Wilk test of normality. Categorical data were represented in percentages and compared by the Chi-square test of association. Normally distributed continuous data were compared using the student t-test.

## Results

The present study included 53 positive culture isolates from a total of 236 samples received from patients with orthopedic implants in the Department of Microbiology of a tertiary care center in Western India. This showed that the culture-positive rate here was 23%.

The clinic demographical details of the patients under study are given in Table 1. The mean age of the patients with culture positivity was 39.47±16.23 years with the maximum number of patients being in the young adult group. There was no significant association between age and gender (p=0.352) but the males of the study group had a younger age of presentation (38.52±16.02 years) than the females (44.11±17.57 years) in the study. An assessment of the comorbidities affecting these patients was also done. The comorbidities along with substance abuse did not show a significant association with the age and gender of the patients (p=0.404, 0.729). The median duration of implants and duration of symptoms were also recorded in the study group. The duration of symptoms was positively and significantly correlated with age with a correlation coefficient of r=0.376 and a p-value of p=0.006. The median duration of symptoms was significantly higher in females (p=0.025). The duration of implant and symptoms in these patients also showed a significant positive correlation where the correlation coefficient 'r' was 0.626 with p<0.001. Patients with diabetes showed an earlier onset of symptoms. Among the 53 samples, 17 (32%) exhibited gram-positive morphology while the remaining 36 (68%) had gram-negative characteristics.

**Table 1:** Clinico-demographic details and microbiological parameters of samples included in the study

Clinico-demographic parameters		N (%) of culture isolates
Age groups (years)	0-20	5 (9.4%)
	21-40	25 (47.2%)
	41-60	16 (30.2%)
	61-80	7 (13.2%)
Gender	Male	44 (83%)
	Female	9 (17%)
Associated comorbidities	Diabetes mellitus	11 (20.8%)
	Hypertension	12 (22.6%)
	Asthma	1 (1.9%)
	Smoking	8 (15.1%)
	Alcohol intake	6 (11.3%)
Site of implant	Tibia	22 (41.5%)
	Femur	19 (35.8%)
	Patella	1 (1.9%)
	Humerus	5 (9.4%)
	Calcaneum	1 (1.9%)
	Radius ulna	5 (9.4%)
Gram stain	Gram-positive	17 (32%)
	Gram-negative	36 (68%)
Biofilm production	No	33 (62.3%)
	Yes	20 (37.7%)
Strength of biofilm	Weak	9 (15.1%)
	Moderate	3 (5.7%)
	Strong	8 (17%)

Table 2 represents the percentage distribution of different isolates along with their characteristic biofilm production. *Staphylococcus aureus* was the only gram-positive bacterium isolated and was also the majority among the isolates. Among the isolates of *Acinetobacter baumannii*, 2 ( 40%) were derived from tibia and femur implants, while the rest originated from radius and ulnar implants. *Escherichia coli* isolates exhibited varied distribution, with 4 (44%) isolated from femur implants, 3 (33%) from tibia implants, and minor percentages from humerus and radius implants. Klebsiella isolates were predominantly found in tibial implants, accounting for 4 cases (44%). As for Staphylococcus aureus, 7 isolates (42%) were obtained from tibia implants, with a similar proportion from femur implants. Notably, tibial implant isolates constituted the majority, which was 22 (42%), followed by femur implants 19 (36%). MRSA isolates formed maximum biofilm over femur implants. Among the only infections from calcaneum, the isolate obtained was klebsiella. Among the biofilm producers, *S. auereus* produced the maximum-strength biofilms. Biofilm production was not significantly associated with age and type of implant, while gender showed a statistically significant association with a male predominance. Patients with diabetes, hypertension, and a history of smoking showed significantly higher chances of biofilm production (p≤0.05).

**Table 2:** The biofilm production and quantification of specific isolates

Names of bacterial isolates	Bacterial isolates based on their biofilm production				
	Isolates producing biofilms			Isolates not producing biofilms	Total number of isolates
	Strong	Moderate	Weak		
*Staphylococcus aureus* (MRSA+MSSA)	5	1	2	9	17
Escherichia coli	0	1	1	7	9
Klebsiella pneumoniae	2	0	2	5	9
Proteus mirabilis	0	0	0	5	5
Morganella morganii	0	0	0	2	2
Pseudomonas aeruginosa	0	1	2	3	6
Acinetobacter baumanii	2	0	1	2	5
Total	9	3	8	33	53

The antibiotic susceptibility analysis is given in Table 3. While most antibiotics showed variable susceptibility, few of the drugs showed complete resistance like penicillin, daptomycin, ofloxacin, quinupristin-dalfopristin, ampicillin, ampicillin/sulbactam, and cefuroxime.

**Table 3:** The antibiotic susceptibility percentage for each of the antibiotics studied in the present study

Antibiotics	Total	Resistant	Susceptible	Intermediate susceptibility	Susceptibility %
Penicillin G	19	19	0	0	0.00
Cefoxitin	17	13	4	0	23.53
Clindamycin	17	11	6	0	35.29
Erythromycin	17	14	3	0	17.65
Azithromycin	17	14	3	0	17.65
Clarithromycin	17	10	3	4	17.65
Vancomycin	17	0	17	0	100.00
Teicoplanin	17	0	17	0	100.00
Co-trimoxazole	38	16	22	0	57.89
Linezolid	17	0	17	0	100.00
Chloramphenicol	35	5	30	0	85.71
Gentamycin	21	5	17	0	80.95
Tetracycline	41	17	23	1	56.10
Doxycycline	42	9	29	4	69.05
Minocycline	38	9	22	6	57.89
Daptomycin	17	17	0	0	0.00
Ciprofloxacin	46	39	7	0	15.22
Levofloxacin	43	33	7	3	16.28
Ofloxacin	17	16	0	1	0.00
Moxifloxacin	17	16	1	0	5.88
Rifampin	17	0	17	0	100.00
Quinupristin-dalfopristin	17	17	0	0	0.00
Ampicillin	17	17	0	0	0.00
Pipercillin	9	7	2	0	22.22
Amoxycilline/claulanic acid	23	17	5	1	21.74
Ampicillin/sulbactam	30	28	0	2	0.00
Piperacilline/tazobactam	34	17	14	3	41.18
Ticarcilline/clavulanic acid	31	24	4	3	12.90
Cefazoline	23	21	2	0	8.70
Cefepime	34	24	10	0	29.41
Ceftraixone	30	24	6	0	20.00
Cefotaxime	26	21	5	0	19.23
Cefoxitin	25	17	8	0	32.00
Cefuroxime	23	23	0	0	0.00
Ceftazidime	25	25	5	0	20.00
Cefixime	25	21	4	0	16.00
Aztreonam	31	19	6	6	19.35
Ertapenem	25	19	6	0	24.00
Imipenem	35	24	6	5	17.14
Meropenem	36	16	17	3	47.22
Gentamycin	32	21	8	3	25.00
Amikacin	33	18	9	6	27.27
Netilimycin	36	16	20	0	55.56
Tobramycin	31	17	5	9	16.13

**Figure 1:**
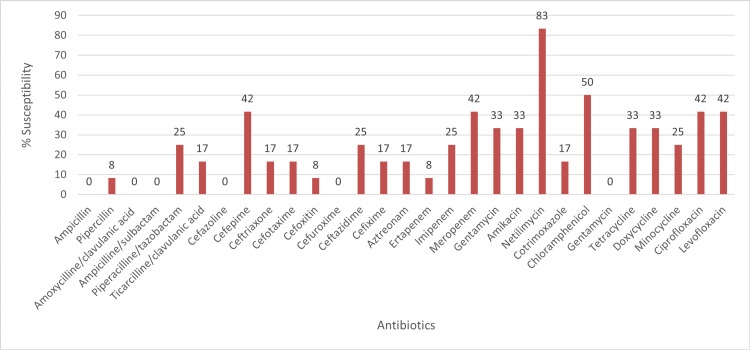
Rates of antibiotic susceptibility in gram-negative biofilm producers

**Figure 2:**
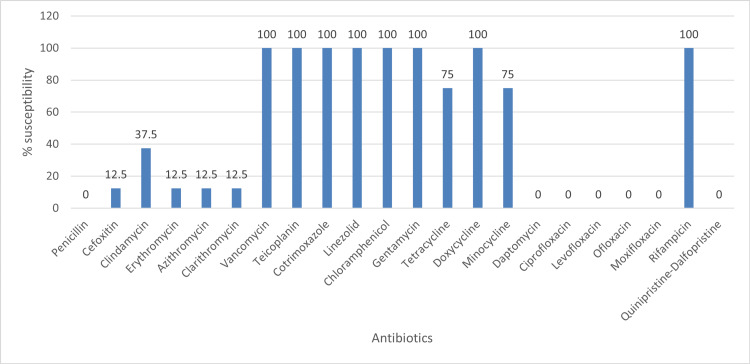
Rates of antibiotic susceptibility in gram-positive biofilm producers

Comparisons among the antibiotic susceptibility pattern (AST) patterns of biofilm producers revealed that resistance rates to certain important antibiotic agents were much higher among biofilm producers than that of their planktonic counterparts. The antibiotic susceptibility patterns of biofilm-forming gram-negative isolate and biofilm-forming gram-positive isolates are shown in Figure 1 and Figure 2, respectively.

## Discussion

ODRI continues to pose a significant challenge in contemporary trauma and orthopedic surgery. This current study on the microbiological profile of orthopedic implants was undertaken because correct information about the most common causative organisms and their antibiotic sensitivity pattern in the setup of a tertiary care hospital is essential for the designing of empirical therapy at the institutional level. The present study analyzed the antibiogram, examined antibiotic susceptibility patterns, and observed in-vitro biofilm production, alongside analyzing their distribution across different patient profiles.

A total of 23% culture positivity rate was found in the present study. The clinical-demographical outcomes of the present study were compared with various previous studies and the details have been presented in Table 4.

**Table 4:** Comparision of present study outcomes with previous studies

Parameters		Present study	Anisha Fernandes et al., 2013 [11]	Benazir et al., 2018 [12]	P. Ganesh Perumal et al., 2001 [13]	M Aditya et al., 2022 [14]	Sagar Dave et al., 2022 [15]	Bernadette G. Pfang et al., 2019 [16]	Roopa Shree S. et al., 2015 [17]	Boyong Wang et.al., 2023 [20]	Mangal Mohammad Naeem et al., 2020 [21]
Year		2024	2013	2018	2001	2022	2022	2019	2015	2023	2020
Site		Gujarat, India	Karnataka, India	Kashmir, India	Karnataka, India	AP, India	Gujarat, India	Madrid, Spain	Karnataka, India	Shanghai, China	Kabul, Afghanistan
Sample size		53	50	100	200	120	150	25	63	44000	30
Total Isolates		53	42	97	200	120	140	482	46	2821	30
Gram stain	Positive	32	54	54	49	65	65	-	56	-	56
	Negative	67	46	46	51	55	35	-	44	-	44
Bacterial isolates	Acinetobacter	9	8	8	-	-	0.03	-	-	-	2.8
	*Escherichia coli*	17	2	2	5	6.6	12	-	-	-	22.2
	Klebsiella pneumoniae	17	9	9	10	8.34	2.6	-	-	-	-
	Proteus mirabilis	9	5	5	2	-	0.02	-	-	-	2.8
	Pseudomonas	11	8	8	11	44.3	12.66	-	-	-	5.5
	Staphylococcus aureus	32	45	45	39	41.57	65.3	-	-	-	50
	Morganella morganii	4	-	-	-	-	0.66	-	-	-	2.8
	Other	-	-	-	-	Enterococcus	-	-	-	-	-
Gender	Male	83	76	79	84.5	Male predominance	78%	-	-	-	86.95%
	Female	17	24	21	15.5	-	22%	-	-	-	13.4 %
Most affected age group (years)		21-40 (25.5%)	Young adult (41%)	20-39 (43% )	30-39 (27%)	Young adult (41%)	-		-	-	-
Site of implant	Tibia	42	16	-	-	21	35.3	-	-	-	-
	Femur	36	26	-	-	37	37.3	-	-	-	-
	Patella	2	-	-	-	-	-	-	-	-	-
	Humerus	9	8	-	-	10	-	-	-	-	-
	Calcaneum/other tarsals	2	7	-	-	11 (foot)	-	-	-	-	-
	Radius ulna	9	5	-	-	11	-	-	-	-	-
	other	-	14 (Both UL+LL)	-	-	10% (knee)	-	-	-	-	-
Comorbidities	Diabetes mellitus	21	-	-	-	-	-	28	-	-	-
	Hypertension	23	-	-	-	-	-	16	-	-	-
	Asthma/steroid intake/immunosuppression	2	-	-	-	-	-	-	5	-	-
	Smoking	15%	-	-	-	-	-	-	-	-	-
	Alcohol intake	11%	-	-	-	-	-	-	-	-	-
	Others	-	-	-	-	-	-	16 (IVD)	-	-	-
Antibiotic discs/pattern of antibiotic susceptibility %	Gram-positive	-	-	-	-	-	-	-	-	-	-
	Penicillin-G	0	-	53	-	-	-	-	-	-	-
	Cefoxitin	23	-	-	-	-	31	-	-	-	-
	Clindamycin	35	-	42	-	75	49.10	-	-	30	-
	Erythromycin	17	-	49.1	-	-	39	-	-	26	-
	Azithromycin	17	-	39.6	-	47	-	-	-	-	-
	Clarithromycin	11	-	-	-	50	38	-	-	-	-
	Vancomycin	100	-	96	-	91.7	60	-	-	100	-
	Teicoplanin	100	-	98	-	-	66.96	-	-	99	-
	Cotrimoxazole	100	-	15	-	50	56	-	-	-	-
	Linezolid	100	-	100	-	72	76.78	-	-	-	-
	Chloramphenicol	100	-	-	-	-	35	-	-	-	-
	Gentamycin	100	-	57	-	-	5	-	-	43	-
	Tetracycline	82	-	66	-	-	40	-	-	65.5	-
	Doxycycline	100	-	-	-	-	-	-	-	-	-
	Ciprofloxacin	0	-	39.6	-	-	25	-	-	57	-
	Ofloxacin	0	-	-	-	41	-	-	-	-	-
	Levofloxacin	0	-	58.5	-	-	15	-	-	58	-
	moxifloxacin	-	-	-	-	-	-	-	-	69	-
	Rifampicin	100	-	-	-	-	60%	-	-	77	-
	Gram-negative	-	-	-	-	-	-	-	-	-	-
	Ampicillin	0	-	-	-	-	-	-	-	-	-
	Piperacillin	8	-	33	-	-	-	-	-	-	-
	Amoxycillin/clavulanic acid	0	-	-	-	14	13	-	-	-	-
	Ampicillin/sulbactam	0	-	-	-	21	-	-	-	-	-
	Piperacillin/tazobactam	25	-	30	-	90	70(avg)	-	-	-	-
	Ticarcillin/clavulanic acid	17	-	31.8	-	-	-	-	-	-	-
	Cefazoline	0	-	-	-	-	-	-	-	-	-
	Cefepime	42	-	27	-	84	30	-	-	-	-
	Ceftriaxone	17	-	27	-	22	30	-	-	-	-
	Cefotaxime	17	-	-	-	-	30	-	-	-	-
	Cefoxitin	8	-	-	-	-	60	-	-	-	-
	Cefuroxime	0	-	-	-	-	-	-	-	-	-
	Ceftazidime	25	-	32	-	27	-	-	-	-	-
	Cefixime	17	-	-	-	10	20	-	-	-	-
	Aztreonam	17	-	-	-	-	-	-	-	-	-
	Ertapenem	8	-	-	-	-	-	-	-	-	-
	Imipenem	25	-	88	-	87	65 avg	-	-	-	-
	Meropenem	42	-	-	-	91	70	-	-	-	-
	Gentamycin	33	-	43	-	45	20	-	-	-	-
	Amikacin	33	-	56.8	-	50	60-80	-	-	-	-
	Netilimycin	83	-	-	-	-	-	-	-	-	-
	Cotrimoxazole	17	-	50	-	21	32	-	-	-	-
	Chloramphenicol	50	-	-	-	-	-	-	-	-	-
	Gentamycin	0	-	43.2	-	-	-	-	-	-	-
	Tetracycline	33	-	-	-	12	35	-	-	-	-
	Doxycycline	33	-	-	-	-	-	-	-	-	-
	Minocycline	25	-	-	-	-	-	-	-	-	-
	Ciprofloxacin	42	-	57	-	38	20	-	-	-	-
	Levofloxacin	42	-	57	-	37	20	-	-	-	-
No. of biofilm-producing isolates/total isolates		20/53		35/97	-	-	-	-	-	-	
Isolates producing biofilm in vitro	Staphylococcus aureus	40	57	57	-	75	-	-	-	-	-
	*Escherichia coli*	10	-	3	-	0	-	-	-	-	-
	Klebsiella pneumoniae	20	-	30	-	16	-	-	-	-	-
	Proteus mirabilis	0	-	0	-	0	-	-	-	-	-
	Morganella morganii	0	-	0	-	0	-	-	-	-	-
	Pseudomonas	15	-	5	-	6	-	-	-	-	-
	Acinetobacter baumannii	15	-	5	-	5	-	-	-	-	-
	Other	-	-	-	-	8.34 (enterococcus)	-	-	-	-	-

In a study by Anisha Fernandes et al. (2013), out of the 50 patients investigated, 42 (84%) had positive cultures [11]. In another study done by Benazir et al. (2018), the culture positivity rate was 86% [12]. The present study had a much lower culture-positive rate, which could be attributed to the time gap and also to the patient cohort, who were given prophylactic and post-surgery antibiotics in the present study.

Analysis of the demographic breakdown revealed a predominant male representation, accounting for 83% of cases, while females comprised the remaining 17%, as seen in various other studies shown in Table 4 [12-15]. The reason could be as simple as males being more involved in road traffic accidents in all parts of the world. The most affected patients were young adults aged 21 to 40 years, as reported by various researchers, highlighting that young adults are more exposed to outdoor activities and more susceptible to road traffic accidents and other incidents [12-15]. The tibia emerged as the most frequent site of infection, accounting for 42% of cases, followed by the femur at 36%. Other studies had a similar incidence of femur implant infection and also reported the femur implant to be the most affected [14,15]. This could be attributed to the fact that lower limbs are most susceptible to serious wounds requiring surgery and are more notorious in healing than upper limb wounds. Comorbidities affected study group patients in all the previous studies of ORDI including our study. While hypertension and diabetes continue to fight for the top spot, substance abuse like alcohol consumption and smoking also contributed to patient morbidity in many patients as indicated in Table 4 [15-17].

Gram staining characters determine an important aspect of pathogen profile with respect to their resistance pattern and formulating empiric and definitive therapy for patient care. The proportion of gram-positive culture isolates was higher compared to gram-negative isolates in most studies as in Table 4 [12-15]. However, a few other studies showed gram-negative isolates as predominant, similar to the findings of the present study, indicating that both sets of microorganisms equally infect patients across the country and the world. In the present study, the most predominant isolate is *Staphylococcus aureus* (32%), followed by *Escherichia* *coli* (17%) and Klebsiella (17%) and rest of the gram-negative non-fermenters like *Acinetobacter baumannii, pseudomonas aeruginosa*, *Proteus mirabilis*, and *Morganella morganii*, which is similar to the study by P. Ganesh Perumal et al. and Sagar Dave et al. [13,15]. Amongst the *Staphylococcus aureus* isolated, 76% are MRSA and 24% are MSSA. This corresponded to the study by P. Ganesh Perumal et al. where MRSA and MSSA were seen in a proportion of 56.8% and 43.2%, respectively [13]. Even in the studies where MSSA was more prevalent, the ratio was not significantly different. This proved that the prevalence of MRSA in ORDI has increased over time leading to difficult hospital stays in most patients. Though the most common gram-negative isolate showed variability, in general, the gram-negative isolates also showed a prominent prevalence in the present and earlier. In our study, a mild percentage of 3.8% were of *Morganella morganii*, which is a rare but well-established cause of orthopedic implant-related infection, congruent to the findings of Konstantinos Anagnostakos et al. and D. Rodrıguez-Pardo et al., which also reported a significant number of Morganella isolates [18,19].

The details of the antibiotic susceptibility comparison are also given in Table 4. The *Staphylococcus aureus* isolates in the present study demonstrated a 100 % susceptibility to teicoplanin and linezolid, cotrimoxazole, tetracycline, and minocycline, similar to isolates reported by Dr. M Aditya et al. and P. Ganesh Perumal et al. [13,14]. In contrast to the present study where betalactam antibiotics like cotrimoxazole have higher susceptibility, P. Ganesh Perumal et al. found that the resistance rate was more pronounced in the beta-lactam antibiotics [13]. Lower susceptibility to erythromycin and azithromycin in the present study compared to the study published by Benazir et al. in 2018 points to increasing patterns of resistance among common bacteria over time [12]. Similarly, an exponential rise in the resistance to fluoroquinolones such as levofloxacin, ciprofloxacin, ofloxacin, and moxifloxacin in the present study compared to earlier studies also indicates a rise in the global concern of antibiotic resistance. While all the study patients were still susceptible to rifampicin, 75% were susceptible to tetracycline and minocycline, and an alarming 100% of the patients were resistant to penicillin.

All the gram-negative isolates were 100% resistant to ampicillin, 25% of isolates showed susceptibility to the commonly used BL-BLI agent piperacillin-tazobactam, while none of them were susceptible to amoxicillin-clavulanate and ticarcillin clavulanic acid. Similar results were reported by M Aditya et al., *Escherichia* *coli* isolates were 100 % sensitive to piperacillin and tazobactam, imipenem and cilastatin, cefepime, and amikacin [14]. Pseudomonas, the predominant isolate was sensitive to Piperacillin tazobactam by 80.7%, imipenem by 76.9%. and imipenem and cilastatin by 80.7%. Lower rates of sensitivities in the same gram-negatives were reported in a later study done by Sagar et al. supporting the claim of rising antibiotic resistance worldwide [15].

In our study, a lower percentage of susceptibility was seen with carbapenems among which meropenem demonstrated the highest susceptibility of 47%, while imipenem and ertapenem showed even lower rates of 25 % and 8 % respectively, indicating a potential increase in Carbapenemase-producing gram-negative bacteria which are also biofilm producers. (CR-GNB). As shown in Table 4, the susceptibility rates were higher in earlier studies, further complimenting the proof of rising widespread antibiotic resistance [20,21].

Following phenotypic identification and susceptibility testing, the present study also tested the isolates for biofilm production using the microtiter plate method. Results showed that biofilm-producing isolates mounted to a significant 37% in the study group taken. *Staphylococcus aureus* was the most predominant biofilm producer (40%) followed by Klebsiella (20%), then Acinetobacter and pseudomonas (15% each), and least by *Escherichia coli* isolates (10%). These results were similar to results reported by M Aditya et al. and Benazir et al. [12,14].

The present study also concluded that male patients were more inclined to be infected with biofilm-producing microorganisms along with patients with a history of diabetes, hypertension, and smoking. 

However, there are certain limitations to the present study such as the duration being only six months, which resulted in a smaller sample size. Certain higher antibiotics like colistin and other synergy tests are not done routinely according to institution laboratory protocol for which a homogenous analysis of such antibiotics is not obtained. Molecular methods of identification were not employed and genetic detection of resistant genes and biofilm-forming genes are to be performed in future undertakings as an extension of current study, which are not currently accomplished in the study.

## Conclusions

This study represents the first interdisciplinary step in a continuous effort to better understand and manage orthopedic implant infections in the hospital. ODRIs are a significant concern due to their chronic and severe nature, leading to increased patient morbidity and surgical failure to restore mobility. Therefore, it is crucial to implement effective management and control strategies through an in-depth analysis of the causative organisms, infection patterns, antibiotic sensitivity rates, and local and institutional factors. Proper infection control measures in operating theaters and inpatient wards are also essential. A detailed microbiological analysis provides the necessary foundation for effective prevention and reduction of the burden of these infections. The study findings revealed that biofilm-producing bacteria are more resistant than their planktonic counterparts, highlighting the importance of studying these bacteria for treating chronic orthopedic implant infections. The choice of empiric antibiotics should be based on local pathogen prevalence and antimicrobial susceptibility patterns, making this study highly significant. Preventive targeted antibiotic therapies for patients with risk factors, such as diabetes, immunosuppression, and smoking, after orthopedic implant surgery, are essential for managing infections in at-risk patients. Given the rising antibiotic resistance, more comprehensive studies with longer follow-up periods are needed to develop effective prevention and treatment protocols for orthopedic implant infections.
